# Bisphenol-A disturbs hormonal levels and testis mitochondrial activity, reducing male fertility

**DOI:** 10.1093/hropen/hoad044

**Published:** 2023-11-15

**Authors:** Do-Yeal Ryu, Won-Ki Pang, Elikanah Olusayo Adegoke, Md Saidur Rahman, Yoo-Jin Park, Myung-Geol Pang

**Affiliations:** Department of Animal Science & Technology and BET Research Institute, Chung-Ang University, Anseong, Republic of Korea; Department of Animal Science & Technology and BET Research Institute, Chung-Ang University, Anseong, Republic of Korea; Department of Animal Science & Technology and BET Research Institute, Chung-Ang University, Anseong, Republic of Korea; Department of Animal Science & Technology and BET Research Institute, Chung-Ang University, Anseong, Republic of Korea; Department of Animal Science & Technology and BET Research Institute, Chung-Ang University, Anseong, Republic of Korea; Department of Animal Science & Technology and BET Research Institute, Chung-Ang University, Anseong, Republic of Korea

**Keywords:** Bisphenol-A, endocrine-disrupting chemicals, male fertility, testis, mitochondrial dynamics, hormonal imbalance

## Abstract

**STUDY QUESTION:**

How does bisphenol-A (BPA) influence male fertility, and which mechanisms are activated following BPA exposure?

**SUMMARY ANSWER:**

BPA exposure causes hormonal disruption and alters mitochondrial dynamics and activity, ultimately leading to decreased male fertility.

**WHAT IS KNOWN ALREADY:**

As public health concerns following BPA exposure are rising globally, there is a need to understand the exact mechanisms of BPA on various diseases. BPA exposure causes hormonal imbalances and affects male fertility by binding the estrogen receptors (ERs), but the mechanism of how it mediates the hormonal dysregulation is yet to be studied.

**STUDY DESIGN, SIZE, DURATION:**

This study consisted of a comparative study using mice that were separated into a control group and a group exposed to the lowest observed adverse effect level (LOAEL) (n = 20 mice/group) after a week of acclimatization to the environment. For this study, the LOAEL established by the US Environmental Protection Agency of 50 mg/kg body weight (BW)/day of BPA was used. The control mice were given corn oil orally. Based on the daily variations in BW, both groups were gavaged every day from 6 to 11 weeks (6-week exposure). Before sampling, mice were stabilized for a week. Then, the testes and spermatozoa of each mouse were collected to investigate the effects of BPA on male fertility. IVF was carried out using the cumulus–oocyte complexes from female hybrid B6D2F1/CrljOri mice (n = 3) between the ages of eight and twelve weeks.

**PARTICIPANTS/MATERIALS, SETTING, METHODS:**

Signaling pathways, apoptosis, and mitochondrial activity/dynamics-related proteins were evaluated by western blotting. ELISA was performed to determine the levels of sex hormones (FSH, LH, and testosterone) in serum. Hematoxylin and eosin staining was used to determine the effects of BPA on histological morphology and stage VII/VIII testicular seminiferous epithelium. Blastocyst formation and cleavage development rate were evaluated using IVF.

**MAIN RESULTS AND THE ROLE OF CHANCE:**

BPA acted by binding to ERs and G protein-coupled receptors and activating the protein kinase A and mitogen-activated protein kinase signaling pathways, leading to aberrant hormone levels and effects on the respiratory chain complex, ATP synthase and protein-related apoptotic pathways in testis mitochondria (*P *<* *0.05). Subsequently, embryo cleavage and blastocyst formation were reduced after the use of affected sperm, and abnormal morphology of seminiferous tubules and stage VII and VIII seminiferous epithelial cells (*P *<* *0.05) was observed. It is noteworthy that histopathological lesions were detected in the testes at the LOAEL dose, even though the mice remained generally healthy and did not exhibit significant changes in BW following BPA exposure. These observations suggest that testicular toxicity is more than a secondary outcome of compromised overall health in the mice due to systemic effects.

**LARGE SCALE DATA:**

Not applicable.

**LIMITATIONS, REASONS FOR CAUTION:**

Since the protein expression levels in the testes were validated, *in vitro* studies in each testicular cell type (Leydig cells, Sertoli cells, and spermatogonial stem cells) would be required to shed further light on the exact mechanism resulting from BPA exposure. Furthermore, the BPA doses employed in this study significantly exceed the typical human exposure levels in real-life scenarios. Consequently, it is imperative to conduct experiments focusing on the effects of BPA concentrations more in line with daily human exposures to comprehensively assess their impact on testicular toxicity and mitochondrial activity.

**WIDER IMPLICATIONS OF THE FINDINGS:**

These findings demonstrate that BPA exposure impacts male fertility by disrupting mitochondrial dynamics and activities in the testes and provides a solid foundation for subsequent investigations into the effects on male reproductive function and fertility following BPA exposure, and the underlying mechanisms responsible for these effects. In addition, these findings suggest that the LOAEL concentration of BPA demonstrates exceptional toxicity, especially when considering its specific impact on the testes and its adverse consequences for male fertility by impairing mitochondrial activity. Therefore, it is plausible to suggest that BPA elicits distinct toxicological responses and mechanistic endpoints based on the particular concentration levels for each target organ.

**STUDY FUNDING/COMPETING INTEREST(S):**

This work was supported by the Basic Science Research Program through the National Research Foundation of Korea (NRF) funded by the Ministry of Education (NRF-2018R1A6A1A03025159). No competing interests are declared.

WHAT DOES THIS MEAN FOR PATIENTS? For decades, concerns have been raised about the impact of environmental hormones, especially bisphenol-A (BPA), on public health and well-being. BPA is widely used in everyday life (for example, in epoxy resin, polycarbonate plastics, and thermal paper dyes) and is known to be associated with male infertility. As a result, various BPA-free products are being introduced and promoted. However, complete replacement of BPA is not feasible, and BPA exposure in daily life is still expected to affect male infertility. The purpose of this study is to raise awareness regarding infertility.

In this study, exposure to high concentrations of BPA did not significantly affect the overall health of mice, including their body weight and kidney histology. However, it did have an impact on mitochondrial activity and led to testicular lesions, resulting in male infertility.

Furthermore, by providing evidence of the mechanisms through which high concentrations of BPA induce infertility, this study may assist in diagnosing male infertility caused by BPA in the future and contribute to the development of treatment methods or therapies.

## Introduction

Over the past decade, concerns about the deleterious effects of endocrine-disrupting chemicals (EDCs) on wildlife and humans have arisen, with the harmful effects of EDCs being confirmed in both scientific and clinical studies (Skinner, 2007; Rahman *et al.*, 2020). Bisphenol-A (BPA) is a prominent and ubiquitous EDC that is found in epoxy resin, polycarbonate plastics, and thermal paper dyes (Beausoleil *et al.*, 2018). Due to its widespread use in consumer products, BPA has the potential to greatly affect the natural environment and public health, with human exposure occurring through oral, inhalation, and transdermal routes via food in BPA-coated packaging, aerosol particles, and BPA-containing skincare products, respectively (Rahman *et al.*, 2021). Large-scale surveys have reported that BPA is detected in the urine of >90% of Americans (Calafat *et al.*, 2008).

Once it has been ingested, BPA has harmful effects on human and animal health through both genomic and non-genomic pathways (Rahman and Pang, 2019). In particular, as an antagonist for both androgen and estrogen receptors (ERs), BPA has been found to damage male fertility by altering hormone receptor binding functions (Rahman *et al.*, 2015), thus disrupting male reproductive functions such as sperm motility/motion kinematics, reproductive organ weights and the expression levels of specific genes associated with male fertility (Rahman *et al.*, 2021). BPA has been shown to interrupt hormonal homeostasis by affecting testicular cells, such as germ, Sertoli, and Leydig cells, resulting in abnormal testicular function. Leydig cells, in particular, produce sex hormones, and an imbalance in these hormone levels can have detrimental effects on male reproduction (Vitku *et al.*, 2015). Abnormal functions of Leydig cells lead to abnormal male phenotypes, secondary sexual male characteristics, dysregulation of specific genes associated with male fertility, and male infertility by disruption of the sex hormone balance. In terms of these aspects, BPA has been arising as a concern due to its detrimental effects on hormone homeostasis and male infertility by mimicking the ERs.

However, the exact mechanisms by which BPA affects male infertility are not fully understood. Oxidative stress has been suggested as a potential mechanism by which BPA impacts male reproduction. Reactive oxygen species (ROS) play a vital role in cellular physiology and can be beneficial for intracellular signaling at optimal levels or deleterious for intracellular signaling when present in excess (Rahman *et al.*, 2021). In particular, it has been reported that disruption of the mitochondrial antioxidant system due to high ROS levels can cause oxidative stress, which affects cellular biomolecules and cell apoptosis. Mitochondria are one of the most critical organelles in male reproduction, being necessary for testosterone production, epigenetic regulation, cell differentiation, and spermatogenesis in the testes (Friedman and Nunnari, 2014; Gao *et al.*, 2016). In addition, mitochondria have a critical role in sperm metabolism as they are important for antioxidant defense systems, calcium regulation, energy production, and sperm apoptosis, which are crucial for sperm motility, capacitation, acrosome reaction, and sperm-oocyte fusion (Boguenet *et al.*, 2021). It has been reported that BPA negatively impacts antioxidant activity, leading to ROS overproduction in rat epididymal sperm (Chitra *et al.*, 2003). Moreover, BPA causes mitochondrial dysfunctions, such as reduced ATP production, mitochondrial mass, and mitochondrial membrane potential (Liu *et al.*, 2013; Wang *et al.*, 2019), which can subsequently affect sperm motility/motion kinematics, biochemical properties, and male fertility (Lin *et al.*, 2013; Wang *et al.*, 2019). While mitochondria are vital to healthy male reproduction, very few studies have been conducted on the effects of BPA on mitochondria in the testes.

Therefore, our study aimed to explore the effects of BPA on the testes and male fertility. We assessed its impact on hormone profiles, ERs, and other proteins associated with key signaling pathways. Additionally, we examined how BPA influences testicular mitochondrial function and the underlying mechanisms. Finally, we observed the stage VII and VIII testicular seminiferous epithelium as well as embryo development after the use of affected sperm, to ascertain whether BPA exposure affects spermatogenesis, fertilization, and embryogenesis. This comprehensive investigation aimed to provide insights into the potential adverse effects of BPA on various physiological processes and reproductive outcomes.

## Materials and methods

### Ethics statement

All procedures were performed according to the standard guidelines for animal studies and were approved by the Institutional Animal Care and Use Committee (IACUC) at Chung-Ang University, Seoul, Korea (IACUC Number: a2022016).

### Chemicals, reagents, and media

Unless otherwise specified, all chemicals and reagents were purchased from Sigma Aldrich (St. Louis, MO, USA). To obtain the objective molecular concentration, BPA was dissolved in corn oil. According to a previously published study, modified Tyrode's medium was used as the basic medium (BM) for spermatozoa (Ryu *et al.*, 2017). Bovine serum albumin (BSA; 4 mg/ml) was added to BM and incubated at 37°C under 5% CO_2_ overnight before analysis.

### Animals and sample collection

Four-week-old CD-1 (ICR) male mice were obtained from Daehan BioLink^®^ (Chungcheongbuk-do, Korea). The mice were housed at 50–60% humidity and 20–25°C under a 12:12 light:dark cycle and fed commercial pellets and water ad libitum. The mice were left to adapt to the environment for a week and divided into control, no observed adverse effect level (NOAEL), and lowest observed adverse effect level (LOAEL) groups (n = 20 mice/group). BPA doses of 5 and 50 mg/kg bw/day were employed as the NOAEL and LOAEL in the present study following U.S. Environmental Protection Agency guidelines (Tyl, 2009; Hengstler *et al.*, 2011). Corn oil with no BPA was administered to the control mice orally. Both groups were gavaged daily from 6 to 11 weeks (i.e. a 6-week treatment) based on daily changes in their body weight (BW). The mice were stabilized for a week before sampling. Afterward, the kidneys, testes, and blood were collected from each mouse. The kidney and testes were stored at −80°C for Western blot analysis and fixed in 4% paraformaldehyde and Bouin's solution overnight for histological analysis, respectively.

### Western blot analysis

Testes were collected from three mice per group and chopped into small (1–2 mm^3^) pieces. Samples were then homogenized in radioimmunoprecipitation assay buffer that contained a complete phosphatase inhibitor and protease inhibitor (Roche Applied Science, Indianapolis, IN, USA). The lysate was centrifuged at 17 000*g* at 4°C for 30 min. The total protein lysates were boiled with sodium dodecyl sulfate (SDS) sample buffer containing 5% β-mercaptoethanol for 5 min at 100°C and loaded onto SDS polyacrylamide gels. Subsequently, electrophoresis was conducted as described in previous studies (Kim and Breton, 2016; Park *et al.*, 2017). The electrophoresed proteins were transferred to polyvinylidene fluoride membranes (Amersham, Piscataway, NJ, USA). The membranes were incubated with a blocking agent for 1 h at room temperature. Antibodies against NADH: ubiquinone oxidoreductase core subunit S2 (NDUFS2), caspase-3 (CAS3), estrogen receptor α (ER α), ATP synthase lipid-binding protein (ATP5A), BCL2-associated X, apoptosis regulator (BAX), tumor protein p53 (p53), B-cell lymphoma 2 (BCL2), estrogen receptor β (ER β) (Santa Cruz), OPA1 (LSBio), cleaved caspase-3 (C.CAS3), protein kinase A (PKA), mitofusin-2 (MFN2), cytochrome C1 (CYC1), NADH: ubiquinone oxidoreductase core subunit V2 (NDUFV2), ATP synthase subunit b, mitochondrial (ATP5F1), mitogen-activated protein kinase 11 (p38), and G protein-coupled receptor (GPCR) (Abcam, Cambridge, UK) were incubated with membranes overnight at 4°C. Then, membranes were washed with PBS-T three times and incubated with horseradish peroxidase (HRP)-conjugated secondary antibodies (Abcam) for 1 h at room temperature. After washing the membranes with PBS-T three times, labeled antigens were produced using enhanced chemiluminescence. ImageJ software (National Institutes of Health, Bethesda, USA) was used to measure the protein levels in each band. The data are presented as the ratio of the protein band intensity to that of beta-actin.

### Immunohistochemistry

The kidneys and testes (n = 3 mice/group) were embedded in paraffin, from which 5-µm-thick sections were collected. As reported previously, the tissue slides were stained using hematoxylin and eosin staining and observed using a fluorescence microscope (Nikon, Tokyo, Japan) (Liu *et al.*, 2013). The assessment of kidney architecture and glomerular injuries was conducted using previously described methods (Kobroob *et al.*, 2018). Seminiferous tubules (STs) with atrophic tubules, germ cell sloughing, and large vacuoles were classified as abnormal, following Dai *et al.* (2017). We also assessed the stage VII and VIII testicular seminiferous epithelium, as described previously (Rahman *et al.*, 2020).

### Serum hormone measurements

The levels of serum FSH, LH, and testosterone (n = 3 mice/group) were measured using commercial assay kits (FSH ELISA Kit, LH ELISA Kit, and Testosterone ELISA Kit, respectively; Novus Biologicals (Centennial, CO, USA)) according to the manufacturer's instructions. Blood samples were collected via cardiac puncture under anesthesia. Serum was collected by centrifugation (4610*g* for 5 min) after incubation at room temperature for 30 min. In brief, the serum samples were added to strip wells, and the antigen subsequently bound to antibodies was colorimetrically quantified at 450 nm using SoftMax Pro 5 software (Molecular Devices, San Jose, CA, USA).

### IVF

Eight- to 12-week-old female hybrid B6D2F1/CrljOri mice were obtained from Nara Biotech (Seoul, Korea) for IVF treatment (n = 3 mice/group). Superovulation was induced via an intraperitoneal injection of pregnant mare serum gonadotropin (PMSG; 5 IU) followed 48 h later by human chorionic gonadotropin (hCG; 5 IU). Cumulus–oocyte complexes (COCs) were collected from the ampulla of the oviduct and transferred to DPBS 15 h after the hCG injection. Before insemination, the COCs were placed in mineral oil submerged in 50 μl of BM supplemented with 10% fetal bovine serum and incubated at 37°C under 5% CO_2_ for 1 h. The COCs were then inseminated with spermatozoa at 1 × 10^6^/ml and incubated at 37°C under 5% CO_2_ for 6 h to induce fertilization (n = 3 mice/group). Following fertilization, the embryos were incubated in 50 μl of BM supplemented with 0.4% BSA under 5% CO_2_ at 37°C. After 18 h, the cleavage rate was measured by counting the number of two-cell embryos compared to the number of zygotes. The two-cell embryos were then transferred to 50 μl of BM supplemented with 0.4% BSA under 5% CO_2_ at 37°C. Five days after insemination, the blastocyst formation rate was measured by counting the number of blastocysts compared to the number of zygotes.

### Statistical analysis

Data were analyzed using student's two-tailed *t*-tests with SPSS 25.0 software (Chicago, IL, USA) to analyze significant differences between the means of the control and BPA LOAEL groups. Differences between the control and BPA groups were considered significant at *P *<* *0.05. Data are presented as the mean ± SD.

## Results

### Effects of NOAEL and LOAEL on histopathological lesion and weight of the testes and kidneys

Histopathological examination of lesions is a crucial and fundamental approach in early toxicological assessments (Crissman *et al.*, 2004). Therefore, to ascertain whether BPA induces lesions at specific concentrations, we examined the morphology of the testis and kidney. Our results revealed that no histopathological lesions were found at NOAEL and LOAEL in kidney. Notably, we observed lesions only at the LOAEL, not at the NOAEL, in the testis (Supplementary Fig. S1). In addition, our results showed that there was no difference in the weight of the testes or kidney relative to BW in the NOAEL- and LOAEL-treated mice when compared to the controls (Supplementary Tables S1 and S2). As a result, this study focused solely on investigating the effects of LOAEL on the testis.

### Effects of BPA on proteins related to regulatory mechanisms in the testes

**Figure 1.**
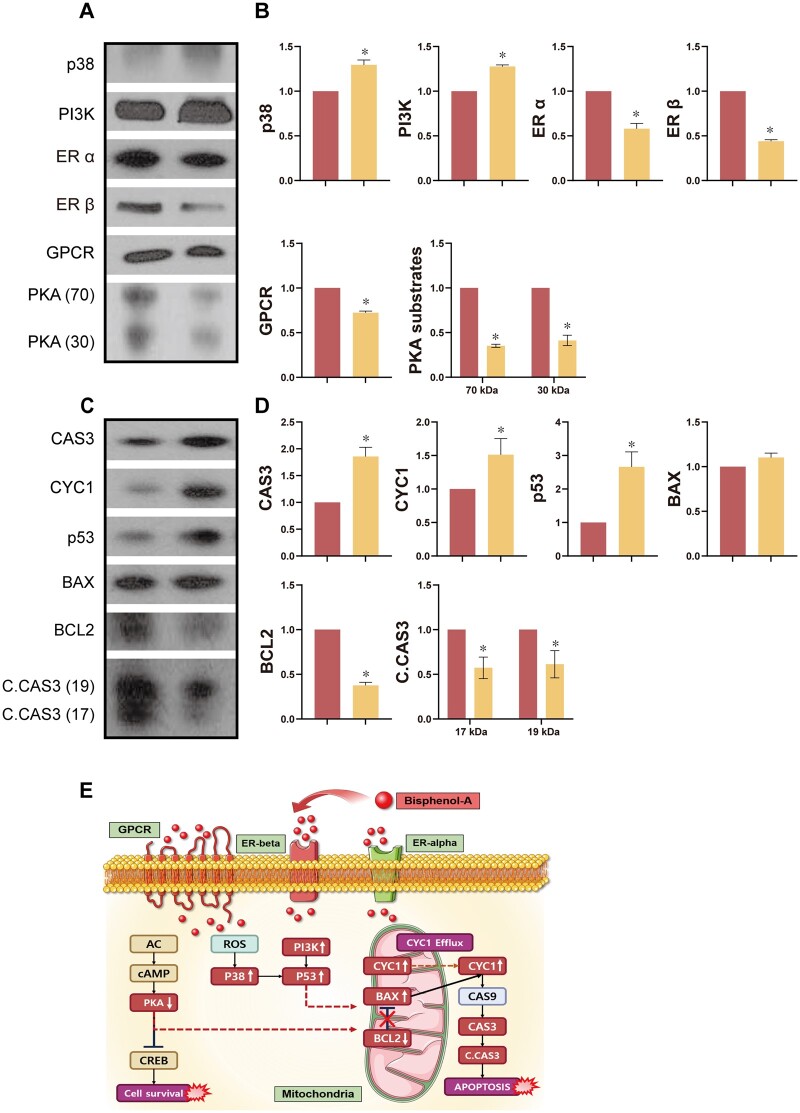
**Effects of Bisphenol-A on signaling and apoptosis-related protein expression levels in testes.** (**A**) Representative Western blot images. (**B**) Levels of signaling proteins in testes. (**C**) Representative Western blot images. (**D**) Levels of apoptosis-related proteins in testes. (**E**) Schematic of estrogenic signaling and apoptosis regulated by Bisphenol-A. Data are mean of three replicates ± SD. Asterisks denote significant differences between control and treatment groups at *P *<* *0.05 by two-tailed Student's *t*-test.

We assessed the levels of proteins related to regulatory mechanisms in the control and BPA-treated testes. Our results showed that ER α and ER β were significantly decreased after BPA exposure, as were the PKA substrates and GPCR (Fig. 1A and B, *P *<* *0.05). We also evaluated the proteins associated with the mitogen-activated protein kinase (MAPK) pathway. Our results showed that levels of p38 and phosphatidylinositol 3-kinase (PI3K) were significantly higher following BPA exposure (Fig. 1A and B, *P *<* *0.05).

### Effects of BPA on apoptotic proteins in the testes

Western blotting was conducted to investigate whether BPA-induced apoptosis occurred in the testes. Our results revealed that CAS3, CYC1, p53, BCL2, and C.CAS3 exhibited significant differences in their expression levels following BPA exposure (Fig. 1C and D, *P *<* *0.05), while there was no difference for BAX.

### Effects of BPA on mitochondrial-related proteins

**Figure 2.**
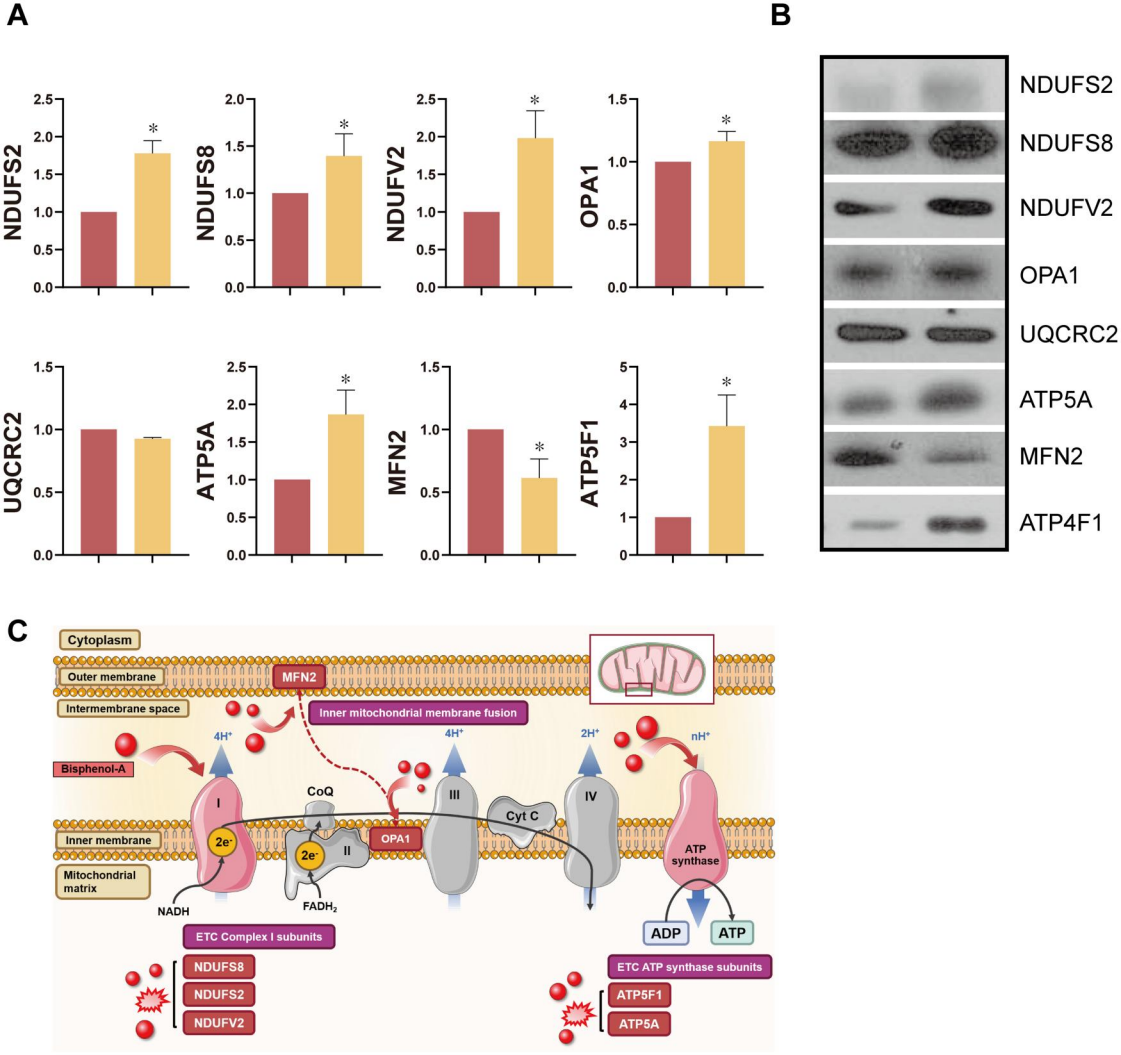
**Effects of Bisphenol-A on the electron transport chain and mitochondrial dynamics-related protein expression levels in testes.** (**A**) Levels of mitochondria-related proteins in testes. (**B**) Representative Western blot images. (**C**) Schematic of mitochondrial dynamics and electron transport chain regulated by Bisphenol-A. Data are mean of three replicates ± SD. Asterisks denote significant differences between control and treatment groups at *P *<* *0.05 by two-tailed Student's *t*-test.

To evaluate the effects of BPA on mitochondrial regulatory mechanisms, we measured the expression levels of UQCRC2, MFN2, OPA1, ATP5A, ATP5F1, NDUFV2, NDUFS2, and NUDFS8 in the testes. We found that BPA exposure affected the levels of mitochondrial fusion proteins, such as MFN2 and OPA1. In addition, levels of electron transport chain-related proteins (ATP5A, ATP5F1, NDUFV2, NDUFS2, and NDUFS8) were significantly higher following BPA exposure (Fig. 2A and B, *P *<* *0.05), except for UQCRC2.

### Effects of BPA on the level of sex hormones

**Figure 3.**
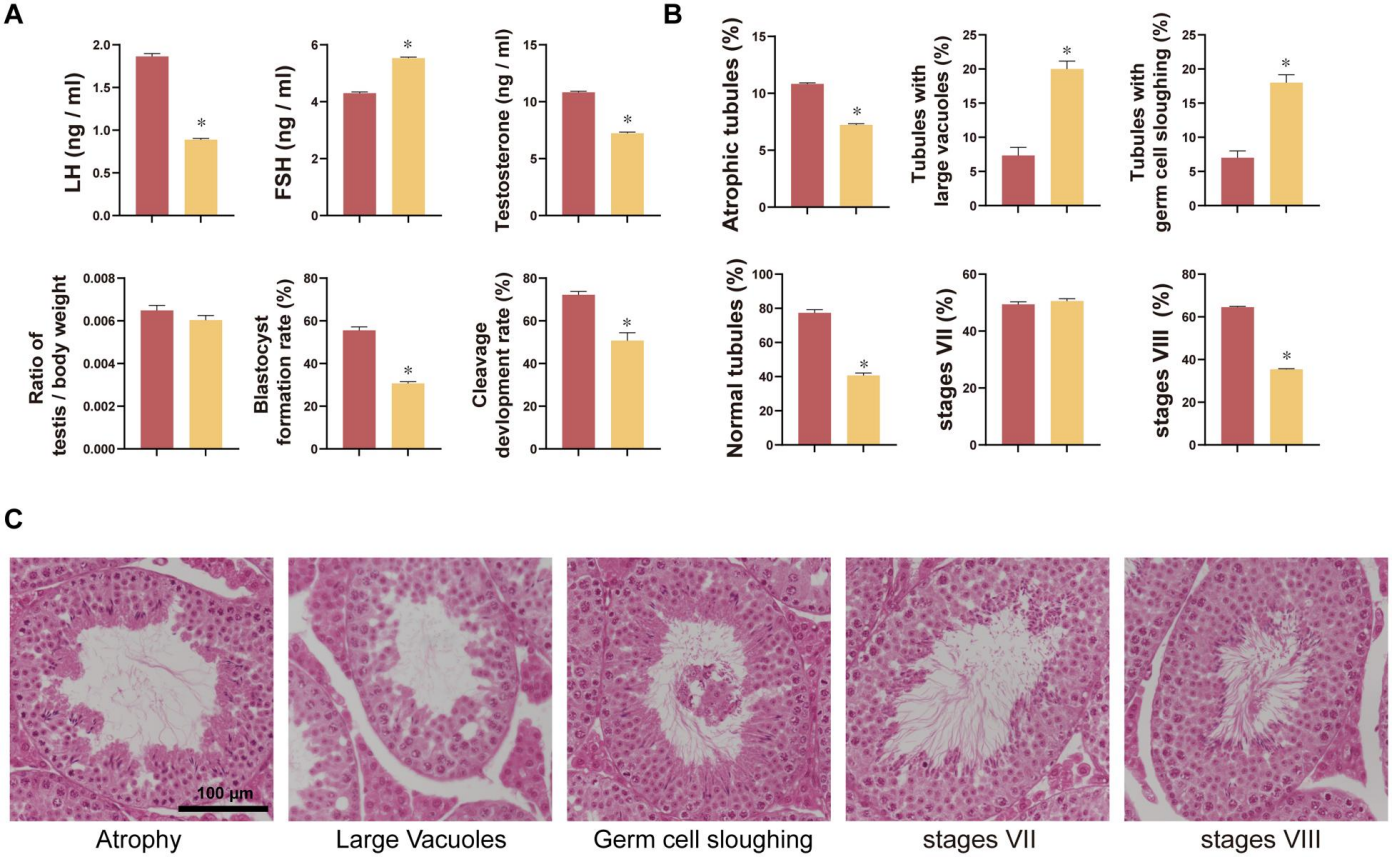
**Effects of Bisphenol-A on testes.** (**A**) Changes in hormone levels, testicular weight relative to body weight, and aspects of male fertility. (**B**) Changes in abnormal testicular morphology and stage VII and VIII seminiferous epithelial cells. (**C**) Representative images of testicular histology. Data are mean of three replicates ± SD. Asterisks denote significant differences between control and treatment groups at *P *<* *0.05 by two-tailed Student's *t*-test. Bar = 100 µm.

To identify the effects of BPA on hormone levels, we performed ELISA assays for FSH, LH, and testosterone. A significant increase in FSH was found in response to BPA exposure, while LH and testosterone levels were significantly lower (Fig. 3A, *P *<* *0.05).

### Effects of BPA on morphology of testes and spermatogenesis

We assessed testicular morphology and spermatogenesis to identify the effects of LOAEL on testicular function and structure. Our results regarding the morphology of the testicular epithelium showed that BPA exposure significantly increased the number of abnormalities, including the tubules with germ cell sloughing and tubules with large vacuoles (Fig. 3B, *P *<* *0.05). Subsequently, we measured the impacts of BPA exposure on testicular spermatogenesis. While a significantly lower number of stage VIII (%) testicular STs was found in response to BPA, there was no difference in the stage VII testicular seminiferous epithelium (Fig. 3B, *P *<* *0.05).

### Effects of BPA on male fertility

Finally, we investigated the fertilization ability of spermatozoa collected from mice exposed to BPA and early embryonic development using an IVF system. As shown in Fig. 3A, BPA exposure significantly decreased the rate of embryo cleavage and blastocyst formation after the use of affected spermatozoa for fertilization (Fig. 3A, *P *<* *0.05).

## Discussion

The LOAEL for BPA, according to US Environmental Protection Agency guidelines, is 50 mg/kg bw/day (Tyl, 2009; Hengstler *et al.*, 2011). However, several studies have reported toxicity at much lower BPA concentrations than the established LOAEL, suggesting that the current LOAEL may be excessively high compared to actual toxic levels (Al-Hiyasat *et al.*, 2002; Akingbemi *et al.*, 2004; Kawai *et al.*, 2007; Jin *et al.*, 2013). Therefore, we evaluated histopathological lesions, examining the toxic effects of BPA on testes and kidneys at NOAEL and LOAEL, to elucidate whether BPA demonstrates varying levels of toxicity in different organs at different concentrations or if it exerts uniform toxicity across all organs at the same concentration. In contrast to other research outcomes, our study findings revealed no discernible kidney toxicity when exposed to either NOAEL or LOAEL of BPA. Only at the LOAEL, we observed pathological testis changes. In addition, even though histopathological lesions were observed in mice exposed to the LOAEL, no alterations in BW or testicular weight were detected, and the animals exhibited a state of apparent health, suggesting that the toxicity at this concentration is primarily localized to the testis. It is tempting to speculate that the divergent outcomes are likely attributable to variations in methodology for BPA exposure, durations, gender, age, and other biological factors inherent to each specific study. However, it is also reasonable to propose that there might be variations in the toxic effects of BPA across different tissues and organs within the organism. Herein, we conducted experiments to elucidate the underlying mechanisms by which the LOAEL concentration, responsible for inducing testicular lesions, exerts its effects.

It has been well-established that BPA binds with ER α and ER β (Delfosse *et al.*, 2012; Kuiper *et al.*, 1998). In addition, BPA acts via both genomic and non-genomic pathways. In the genomic pathway, BPA offers a ligand for cytoplasm ERs or nucleus ERs. Binding with these ERs impacts the characteristics of nuclear chromatin and regulates transcriptional/translational processes, thus disrupting cell proliferation, differentiation, and survival (Heldring *et al.*, 2007; Rahman and Pang, 2019). BPA also binds to GPCR 30 in testicular cells through a non-genomic signaling pathway, which leads to the rapid phosphorylation of PKA, MAPK, and PI3K and affects the levels of protein kinase C, adenosine monophosphate and intracellular calcium, thus seriously damaging cells and tissue (Kitamura *et al.*, 2005; Williams *et al.*, 2014). Regarding male reproduction, Williams *et al.* reported that BPA binds with ERs in the testes and has a harmful effect on gametogenesis and steroidogenesis during adulthood (Li *et al.*, 2009; Williams *et al.*, 2014; Goncalves *et al.*, 2018). In Leydig cells, both low and high doses of BPA reduce cell viability, mitochondrial membrane potential, and testosterone production by inactivating ER α (Goncalves *et al.*, 2018; Wang *et al.*, 2019). In another study, Polash *et al.* found that BPA impairs functional and physiological germ cells (Karmakar *et al.*, 2020). Consistent with these studies, our results showed that ERs were significantly negatively impacted by BPA exposure, leading to the significant induction of phosphor-MAPK (p38), phosphor-tyrosine, and PKA substrates in the testes, thus activating kinase pathways. It has been shown that p38 can be tyrosine-phosphorylated in response to extracellular stimuli, subsequently activating the kinase system (Zarubin and Han, 2005). It has also been reported that the PKA and MAPK signaling pathways play a vital role in steroidogenesis in Leydig cells, with crosstalk between the two (Gerits *et al.*, 2008; Barbagallo *et al.*, 2020; Li *et al.*, 2020). Consistent with previous findings, our results show that BPA has effects by binding with ERs and by activating MAPK and PKA signaling, consequently affecting both testicular function and male fertility. In addition, binding to ERs may affect the regulation of gene-expression-associated signaling pathways, resulting in disruption of testicular function.

Leydig cells produce testosterone, which is critical for secondary sexual characteristics and spermatogenesis in the mitochondria (Mathur and D'Cruz, 2011). Testosterone is produced in response to LH. It has been reported that LH binding to the receptors on Leydig cells activates adenylyl cyclase and G protein, thus increasing cyclic AMP (cAMP) levels (Dufau, 1988). cAMP consequently enhances cholesterol transport to the inner mitochondrial membrane, leading to the synthesis of testosterone. In addition, the production of FSH is controlled by testosterone levels and inhibin produced by the testes. Although several studies have shown that BPA exposure is associated with changes in hormone levels, the patterns of these changes have been inconsistent between these studies (Galloway *et al.*, 2010; LaRocca *et al.*, 2011; Xi *et al.*, 2011; Kobayashi *et al.*, 2012; Zhou *et al.*, 2013). In this study, BPA exposure significantly decreased the LH and testosterone levels and increased FSH levels. Based on these findings, it is assumed that BPA suppressed the levels of testosterone in conjunction with the lower LH, thus increasing the FSH levels. In addition, our results demonstrated that PKA substrates were significantly lower following BPA exposure. Therefore, it is assumed that BPA binds with LH receptors on Leydig cells, subsequently decreasing cAMP production and interrupting testosterone production, which leads to the failure of spermatogenesis and consequent male infertility.

We also evaluated the effects of BPA exposure on testicular mitochondria. As mentioned above, mitochondria play a crucial role in the testes via testosterone synthesis and ATP production, which are important for spermatogenesis and sperm flagella (Boussouar and Benahmed, 2004; Park and Pang, 2021). Changyun *et al.* revealed that BPA exposure is associated with ATP deficiency in dead cells. Another study reported that the activity of the mitochondrial respiratory chain complex and intracellular ATP levels were significantly lower following BPA exposure in the liver and colon (Wang *et al.*, 2019; Xiao *et al.*, 2019). In terms of male reproduction, BPA induces aberrations in the mitochondria by decreasing ATP levels and the mitochondrial membrane potential, which can affect sperm motility and production (Chitra *et al.*, 2003; Chattopadhyay *et al.*, 2010). BPA also affects testicular cells through ROS-induced damage and cell apoptosis by activating apoptotic signaling (Murata and Kang, 2018).

Therefore, we monitored the levels of mitochondrial respiratory chain complex I (NDUFS2 and NDUFV2), mitochondrial ATP synthase (ATP5A and ATP5F1), mitochondrial fusion enzymes (MFN2 and OPA1), and several apoptosis markers (BAX, BCL2, CYC1, CAS3, Cleaved CAS3, and p53) to investigate the effects of BPA on testicular mitochondria. Our results demonstrated that BPA exposure significantly increased NDUFV2, NDUFS2, NDUFS8, ATP5F1, and ATP5A. There are two possibilities to explain these results. First, cells with lower ATP production due to BPA exposure could have higher expression levels of proteins related to ATP synthesis to overcome the deficiency. The other possibility is that the respiratory chain complex-related proteins are overexpressed to reduce the excessive ROS levels induced by BPA. In addition, MFN2 and OPA1 are essential proteins for mitochondrial fusion, helping to regulate mitochondrial homeostasis and circuitry. It has been reported that BPA exposure affects mitochondria fusion without affecting MFN2 and OPA1. However, in contrast with these previous findings, we found that BPA exposure affected the expression levels of MFN2 and OPA1 (Agarwal *et al.*, 2016), suggesting that BPA does have an impact on mitochondrial fusion in mice.

Subsequently, we evaluated the expression levels of apoptosis markers in response to BPA exposure. BPA impacts ROS-mediated damage and cell apoptosis by activating apoptotic signaling. Our results showed that the apoptosis markers CYC1, CAS3, C.CAS3, and p53, were significantly influenced by BPA exposure. Thus, BPA may influence cell apoptosis through mitochondria, resulting in the degeneration of testicular mitochondrial function via the p53 apoptotic pathway. It is thus plausible to suggest that BPA exposure might disturb mitochondrial dynamics, meaning that insufficient energy is produced for cell activity, leading to various dysfunctions, including male fertility, via the apoptosis pathways in mitochondria. However, few studies have investigated mitochondrial function in the testes following BPA exposure. Therefore, further research is required to understand the underlying mechanisms affecting mitochondrial activity following BPA exposure.

Testicular histology is vital in assessing male reproductive disorders (Russell *et al.*, 2002). It has been reported that various layers of germ cells can be categorized into recognizable arrangements in STs, which are referred to as seminiferous epithelium stages (Meistrich and Hess, 2013). We found that the number of abnormal STs, such as tubules with large vacuoles and germ cell sloughing, was significantly higher following BPA exposure, which may result in lower overall sperm function and levels. Furthermore, the number of stage VIII seminiferous epithelial cells was significantly lower following BPA exposure in the testes. It has been reported that BPA induces germ cell apoptosis, especially at stages VII and VIII (D'Souza *et al.*, 2005). Although the number of stage VII seminiferous epithelial cells did not significantly change, the number of stage VIII seminiferous epithelial cells dramatically decreased. Our findings point to a possible delay in spermiation at stage VIII following BPA exposure (Liu *et al.*, 2014). Thus, BPA exposure may negatively impact spermatogenesis, leading to lower sperm numbers and decreased motility (Rahman *et al.*, 2021). Likewise, another important finding from the current study was that the spermatozoa from testes exposed to BPA caused a significantly lower rate of embryo cleavage and development compared to the control group, likely as a consequence of damaged testicular function. Therefore, it is conceivable that BPA leads to cell apoptosis by disruption of testis mitochondrial activity resulting in an imbalance of sex hormones, abnormal testicular morphology, and decreased male fertility.

## Conclusion

In numerous studies, lower concentrations of BPA than the LOAEL have been reported to elicit toxicity. However, our research revealed that BPA induces toxicity at different concentrations in each respective organ, with LOAEL showing significant toxicity in the testes. It is plausible that BPA induces toxicity with a higher sensitivity specifically in the testes of mouse suggesting that the toxicity of the testis is not a secondary consequence of unhealthy mice resulting from systemic effects. Nonetheless, the concentrations employed in this study greatly exceed the levels of BPA typically encountered by the general human population. Consequently, it is imperative to undertake further research to establish environmentally relevant exposure concentrations and subsequently conduct studies to evaluate the impact of such doses on testicular function and mitochondrial activity. Furthermore, conducting investigations that encompass the impact of environmentally pertinent BPA doses on various tissues and organs within the organism would contribute to a comprehensive comprehension of the toxicity of BPA and its potential repercussions for overall health and well-being.

To our knowledge, this is the first study to investigate the effects of BPA exposure on male fertility by determining the alteration of mitochondrial protein expression levels in mouse testis. In the present study, we demonstrated that BPA acts by binding to ERs and by activating PKA and MAPK signaling, which disrupts the hormone balance and subsequently male fertility. In addition, the signaling cascades activated by BPA influence the respiratory chain complex, ATP synthase, and protein-related apoptotic pathways of testis mitochondria. Subsequently, higher levels of apoptosis may play an important role in the morphology of STs and stage VII and VIII seminiferous epithelial cells, thus reducing male fertility. Therefore, our findings suggest that testicular mitochondrial proteins and activities may be helpful biomarkers for diagnosing male reproductive disorders following BPA exposure. However, further research is required to thoroughly define the functions and mechanisms of precise BPA exposures on specific testicular cell types, such as Leydig, Sertoli, and germ cells for a better understanding of how BPA processes affect the testicular mitochondrial proteins.

## Supplementary Material

hoad044_Supplementary_Data

## Data Availability

All data are incorporated into the article and its online supplementary material.
