# Supplementary material for: Bisphenol-A disturbs hormonal levels and testis mitochondrial activity, reducing male fertility
Source: Hum Reprod Open. 2023 Nov 15;2023(4):hoad044. doi: 10.1093/hropen/hoad044 (PMC10681812; doi:10.1093/hropen/hoad044)
Supplement: hoad044_Supplementary_Data [file hoad044_supplementary_data.zip › HRO-23-0183-R2-SuppTables1-2_EO.docx]

**Supplementary tables**

**Bisphenol-A disturbs hormonal levels and testis mitochondrial activity, reducing male fertility**

Do-Yeal Ryu, Won-Ki Pang, Elikanah Olusayo Adegoke, Md Saidur Rahman, Yoo-Jin Park, and Myung-Geol Pang*

*Department of Animal Science & Technology and BET Research Institute,* *Chung-Ang University, Anseong, Gyeonggi-do 17546, Republic of Korea*

*Corresponding author:

*Department of Animal Science & Technology and BET Research Institute, Chung-Ang University, Anseong, Gyeonggi-do 17546, Republic of Korea*

*[mgpang@cau.ac.kr](mailto:mgpang@cau.ac.kr) (M.-G. Pang)*

*Tel: +82.31.670.4841*

*Cell: +82.10.9003.1281*

Supplementary Table S1. The effects of Bisphenol-A on ratio of testis / body weight

Supplementary Table S2. The effects of Bisphenol-A on ratio of kidney / body weight

**Supplementary Table S1.** **The effects of** Bisphenol-A **on ratio of testis / body weight**

| **Control** |  |  |  | **NOAEL** |  |  |  | **LOAEL** |  |  |  |
| --- | --- | --- | --- | --- | --- | --- | --- | --- | --- | --- | --- |
| **Mice No.** | **Body Weight (g)** | **Testis Weight (g)** | **Ratio** | **Mice No.** | **Body Weight (g)** | **Testis Weight (g)** | **Ratio** | **Mice No.** | **Body Weight (g)** | **Testis Weight (g)** | **Ratio** |
| 1 | 39.02 | 0.26 | 0.0067 | 1 | 37.54 | 0.23 | 0.0061 | 1 | 39.31 | 0.24 | 0.0061 |
| 2 | 35.41 | 0.24 | 0.0068 | 2 | 36.66 | 0.25 | 0.0068 | 2 | 38.02 | 0.24 | 0.0063 |
| 3 | 41.63 | 0.25 | 0.0060 | 3 | 38.64 | 0.24 | 0.0062 | 3 | 39.47 | 0.24 | 0.0061 |
| 4 | 40.52 | 0.24 | 0.0059 | 4 | 35.78 | 0.24 | 0.0067 | 4 | 39.55 | 0.23 | 0.0058 |
| 5 | 35.91 | 0.24 | 0.0067 | 5 | 36.66 | 0.22 | 0.0060 | 5 | 37.67 | 0.25 | 0.0066 |
| 6 | 39.96 | 0.23 | 0.0058 | 6 | 36.67 | 0.22 | 0.0060 | 6 | 40.67 | 0.25 | 0.0061 |
| 7 | 38.87 | 0.24 | 0.0062 | 7 | 38.27 | 0.24 | 0.0063 | 7 | 41.53 | 0.26 | 0.0063 |
| 8 | 37.98 | 0.24 | 0.0063 | 8 | 39.77 | 0.26 | 0.0065 | 8 | 38.10 | 0.22 | 0.0058 |
| 9 | 37.47 | 0.21 | 0.0056 | 9 | 38.64 | 0.23 | 0.0060 | 9 | 36.18 | 0.21 | 0.0058 |
| 10 | 36.92 | 0.21 | 0.0057 | 10 | 36.06 | 0.25 | 0.0069 | 10 | 39.07 | 0.24 | 0.0061 |
| 11 | 39.61 | 0.26 | 0.0066 | 11 | 37.45 | 0.25 | 0.0067 | 11 | 36.44 | 0.22 | 0.0060 |
| 12 | 37.59 | 0.23 | 0.0061 | 12 | 43.35 | 0.27 | 0.0062 | 12 | 36.65 | 0.26 | 0.0071 |
| 13 | 35.90 | 0.24 | 0.0067 | 13 | 39.19 | 0.24 | 0.0061 | 13 | 39.46 | 0.25 | 0.0063 |
| 14 | 35.55 | 0.23 | 0.0065 | 14 | 37.14 | 0.26 | 0.0070 | 14 | 34.77 | 0.23 | 0.0066 |
| 15 | 39.61 | 0.24 | 0.0061 | 15 | 40.95 | 0.27 | 0.0066 | 15 | 37.79 | 0.24 | 0.0064 |
| 16 | 36.17 | 0.24 | 0.0066 | 16 | 39.62 | 0.24 | 0.0061 | 16 | 35.67 | 0.20 | 0.0056 |
| 17 | 39.05 | 0.24 | 0.0061 | 17 | 38.38 | 0.23 | 0.0060 | 17 | 38.71 | 0.24 | 0.0062 |
| 18 | 36.92 | 0.24 | 0.0065 | 18 | 39.53 | 0.21 | 0.0053 | 18 | 38.19 | 0.24 | 0.0063 |
| 19 | 37.59 | 0.25 | 0.0067 | 19 | 39.60 | 0.25 | 0.0063 | 19 | 37.77 | 0.25 | 0.0066 |
| 20 | 41.57 | 0.26 | 0.0063 | 20 | 37.45 | 0.24 | 0.0064 | 20 | 36.38 | 0.25 | 0.0069 |
| Average | 38.16 | 0.239 | 0.0063 | Average | 38.37 | 0.242 | 0.0063 | Average | 38.07 | 0.238 | 0.0063 |

NOAEL: No observed adverse effect level. LOAEL: Lowest observed adverse effect level.

**Supplementary Table S2.** **The effects of** Bisphenol-A **on ratio of kidney / body weight**

| **Control** |  |  |  | **NOAEL** |  |  |  | **LOAEL** |  |  |  |
| --- | --- | --- | --- | --- | --- | --- | --- | --- | --- | --- | --- |
| **Mice No.** | **Body Weight (g)** | **Kidney Weight (g)** | **Ratio** | **Mice No.** | **Body Weight (g)** | **Kidney Weight (g)** | **Ratio** | **Mice No.** | **Body Weight (g)** | **Kidney Weight (g)** | **Ratio** |
| 1 | 39.02 | 0.40 | 0.0103 | 1 | 37.54 | 0.38 | 0.0101 | 1 | 39.31 | 0.40 | 0.0102 |
| 2 | 35.41 | 0.39 | 0.0110 | 2 | 36.66 | 0.37 | 0.0101 | 2 | 38.02 | 0.38 | 0.0100 |
| 3 | 41.63 | 0.40 | 0.0096 | 3 | 38.64 | 0.39 | 0.0101 | 3 | 39.47 | 0.39 | 0.0099 |
| 4 | 40.52 | 0.40 | 0.0099 | 4 | 35.78 | 0.37 | 0.0103 | 4 | 39.55 | 0.37 | 0.0094 |
| 5 | 35.91 | 0.39 | 0.0109 | 5 | 36.66 | 0.39 | 0.0106 | 5 | 37.67 | 0.37 | 0.0098 |
| 6 | 39.96 | 0.42 | 0.0105 | 6 | 36.67 | 0.38 | 0.0104 | 6 | 40.67 | 0.39 | 0.0096 |
| 7 | 38.87 | 0.38 | 0.0098 | 7 | 38.27 | 0.39 | 0.0102 | 7 | 41.53 | 0.44 | 0.0106 |
| 8 | 37.98 | 0.37 | 0.0097 | 8 | 39.77 | 0.40 | 0.0101 | 8 | 38.10 | 0.39 | 0.0102 |
| 9 | 37.47 | 0.40 | 0.0107 | 9 | 38.64 | 0.40 | 0.0104 | 9 | 36.18 | 0.36 | 0.0100 |
| 10 | 36.92 | 0.36 | 0.0098 | 10 | 36.06 | 0.38 | 0.0105 | 10 | 39.07 | 0.38 | 0.0097 |
| 11 | 39.61 | 0.38 | 0.0096 | 11 | 37.45 | 0.36 | 0.0096 | 11 | 36.44 | 0.37 | 0.0102 |
| 12 | 37.59 | 0.4 | 0.0106 | 12 | 43.35 | 0.42 | 0.0097 | 12 | 36.65 | 0.36 | 0.0098 |
| 13 | 35.90 | 0.35 | 0.0097 | 13 | 39.19 | 0.36 | 0.0092 | 13 | 39.46 | 0.37 | 0.0094 |
| 14 | 35.55 | 0.36 | 0.0101 | 14 | 37.14 | 0.37 | 0.0100 | 14 | 34.77 | 0.36 | 0.0104 |
| 15 | 39.61 | 0.39 | 0.0098 | 15 | 40.95 | 0.36 | 0.0088 | 15 | 37.79 | 0.36 | 0.0095 |
| 16 | 36.17 | 0.38 | 0.0105 | 16 | 39.62 | 0.41 | 0.0103 | 16 | 35.67 | 0.35 | 0.0098 |
| 17 | 39.05 | 0.40 | 0.0102 | 17 | 38.38 | 0.39 | 0.0102 | 17 | 38.71 | 0.40 | 0.0103 |
| 18 | 36.92 | 0.37 | 0.0100 | 18 | 39.53 | 0.39 | 0.0099 | 18 | 38.19 | 0.39 | 0.0102 |
| 19 | 37.59 | 0.36 | 0.0096 | 19 | 39.60 | 0.39 | 0.0098 | 19 | 37.77 | 0.40 | 0.0106 |
| 20 | 41.57 | 0.42 | 0.0101 | 20 | 37.45 | 0.38 | 0.0101 | 20 | 36.38 | 0.37 | 0.0102 |
| Average | 38.16 | 0.386 | 0.0101 | Average | 38.37 | 0.384 | 0.0100 | Average | 38.07 | 0.380 | 0.0100 |

NOAEL: No observed adverse effect level. LOAEL: Lowest observed adverse effect level.
