# Supplementary material for: Bisphenol-A disturbs hormonal levels and testis mitochondrial activity, reducing male fertility
Source: Hum Reprod Open. 2023 Nov 15;2023(4):hoad044. doi: 10.1093/hropen/hoad044 (PMC10681812; doi:10.1093/hropen/hoad044)
Supplement: hoad044_Supplementary_Data [file hoad044_supplementary_data.zip › Supplementary_Figures_EO.docx]

**Supplementary Figure**

**Bisphenol-A disturbs hormonal levels and testis mitochondrial activity, reducing male fertility**

Do-Yeal Ryu, Won-Ki Pang, Elikanah Olusayo Adegoke, Md Saidur Rahman, Yoo-Jin Park, and Myung-Geol Pang*

*Department of Animal Science & Technology and BET Research Institute,* *Chung-Ang University, Anseong, Gyeonggi-do 17546, Republic of Korea*

*Corresponding author:

*Department of Animal Science & Technology and BET Research Institute, Chung-Ang University, Anseong, Gyeonggi-do 17546, Republic of Korea*

*[mgpang@cau.ac.kr](mailto:mgpang@cau.ac.kr) (M.-G. Pang)*

*Tel: +82.31.670.4841*

*Cell: +82.10.9003.1281*

Supplementary Figure S1. Representative images of testis and kidney histology following BPA exposure.

**
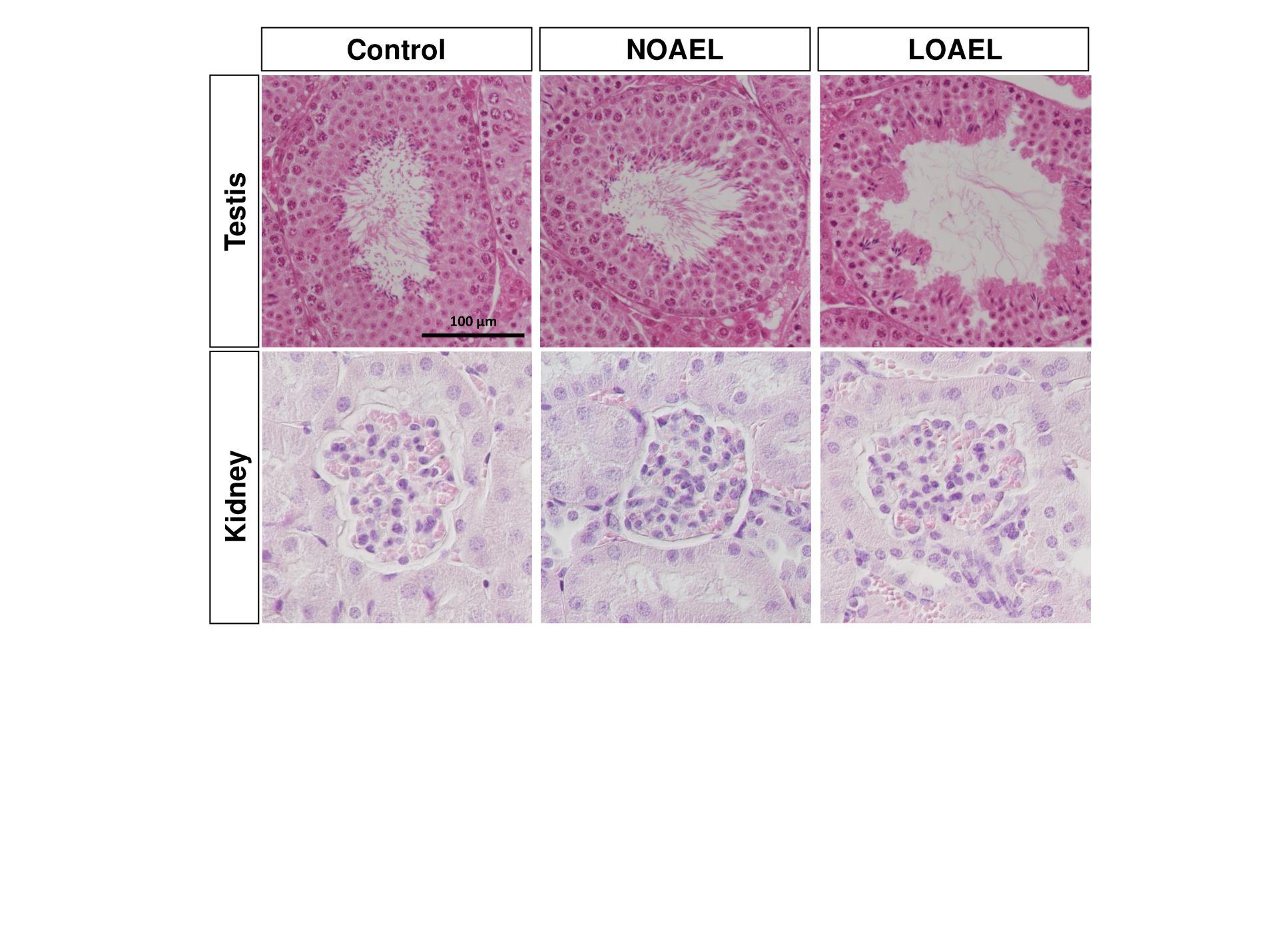
**

**Supplementary Figure S1. Representative images of testis and kidney histology following NOAEL and LOAEL exposure. NOAEL = No observed adverse effect level, LOAEL = Lowest observed adverse effect level. Bar = 100 µm**
